# Butyrolactone-I from Marine Fungal Metabolites Mitigates Heat-Stress-Induced Apoptosis in IPEC-J2 Cells and Mice Through the ROS/PERK/CHOP Signaling Pathway

**DOI:** 10.3390/md22120564

**Published:** 2024-12-17

**Authors:** Xueting Niu, Shengwei Chen, Xinchen Wang, Jiaying Wen, Xiaoxi Liu, Yanhong Yong, Zhichao Yu, Xingbing Ma, A. M. Abd El-Aty, Xianghong Ju

**Affiliations:** 1 Marine Medical Research and Development Centre, Shenzhen Institute of Guangdong Ocean University, Shenzhen 518120, China; nxt1208@163.com (X.N.); csw9610@163.com (S.C.); wxc1202@outlook.com (X.W.); 18718018181@139.com (J.W.); 2 College of Coastal Agricultural Sciences, Guangdong Ocean University, Zhanjiang 524088, China; liuxiaoxi_06@163.com (X.L.); yongyanhong-007@163.com (Y.Y.); yujingmary@163.com (Z.Y.); mxb1984612@126.com (X.M.); 3 Department of Pharmacology, Faculty of Veterinary Medicine, Cairo University, Giza 12211, Egypt; abdelaty44@hotmail.com; 4 Department of Medical Pharmacology, Medical Faculty, Ataturk University, Erzurum 25240, Turkey

**Keywords:** Butyrolactone-I (BTL-I), heat stress, intestinal apoptosis, HSP70 and HSP90 expression, oxidative stress, endoplasmic reticulum stress

## Abstract

Heat stress poses a significant challenge to animal husbandry, contributing to oxidative stress, intestinal mucosal injury, and apoptosis, which severely impact animal health, growth, and production efficiency. The development of safe, sustainable, and naturally derived solutions to mitigate these effects is critical for advancing sustainable agricultural practices. Butyrolactone-I (BTL-I), a bioactive compound derived from deep-sea fungi (Aspergillus), shows promise as a functional feed additive to combat heat stress in animals. This study explored the protective effects of BTL-I against heat-stress-induced oxidative stress and apoptosis in IPEC-J2 cells and mice. Our findings demonstrated that BTL-I effectively inhibited the heat-stress-induced upregulation of HSP70 and HSP90, alleviating intestinal heat stress. Both in vitro and in vivo experiments revealed that heat stress increased intestinal cell apoptosis, with a significant upregulation of Bax/Bcl-2 expression, while BTL-I pretreatment significantly reduced apoptosis-related protein levels, showcasing its protective effects. Furthermore, BTL-I suppressed oxidative stress markers (ROS and MDA) while enhancing antioxidant activity (SOD levels). BTL-I also reduced the expression of p-PERK, p-eIF2α, ATF4, and CHOP, mitigating oxidative and endoplasmic reticulum stress in intestinal cells. In conclusion, BTL-I demonstrates the potential to improve animal resilience to heat stress, supporting sustainable livestock production systems. Its application as a natural, eco-friendly feed additive will contribute to the development of sustainable agricultural practices.

## 1. Introduction

As the global climate is continuously changing, high temperatures and humidity pose significant challenges to animal production. Within a suitable environmental range, heat production and heat dissipation are balanced and the physiological and metabolic conditions are good. When the ambient temperature exceeds a suitable range, the body’s heat dissipation system becomes imbalanced, resulting in the accumulation of excessive heat within the body. This leads to a nonspecific physiological response called heat stress (HS). HS is a crucial factor that induces stress in tropical and subtropical regions, causing severe oxidative stress [1], damage to the intestinal mucosa [2,3], inflammatory responses [4], and apoptosis [5]. These may be among the key factors causing a reduced feed intake, decreased milk and egg production, and weight loss [6].

The intestine is an important digestive organ that absorbs nutrients and excretes metabolic waste [7]. Digested food is broken down into nutrients in the small intestine and enters the bloodstream through absorption by intestinal epithelial cells. Moreover, the intestinal barrier protects the host from harmful bacteria. Under heat stress, the intestinal blood flow is reduced, and hypoxia results in ATP depletion, decreased ion pump activity and cell activity, and cell membrane and tight junction damage; thus, many pathogenic microorganisms invade and cause disease [8]. Heat stress can cause severe cell apoptosis and tissue damage [3,9], which are important causes of heat stress pathogenesis. Research has demonstrated that heat stress (HS) can accumulate reactive oxygen species and impair mitochondrial function, thus inducing apoptosis [10]. Moreover, HS has been shown to induce mammary epithelial cell apoptosis through an ROS-independent pathway [11]. Acute HS can activate p53-mediated mitochondrial apoptosis, contributing to liver injury [12,13]. Another study reported that acute heat stress triggers the unfolded protein response (UPR), leading to endoplasmic reticulum stress and subsequent apoptosis in the liver [14,15]. These findings underscore the close relationship between apoptosis and HS.

Butyrolactone-I (BTL-I) is derived from the deep-sea fungus Aspergillus C23-3, an endophytic fungus that was isolated from Pukou coral (Porites pugorica) in Xuwen (Guangdong, China). Extensive research has highlighted its potential as a potent α-glucosidase inhibitor, offering beneficial effects in managing type 2 diabetes by modulating gut microbes [16]. Additionally, BTL-I serves as a CDK-2 kinase inhibitor, promoting the apoptosis of cancer cells while exerting a protective effect against neuronal apoptosis [17]. However, whether BTL-I has the potential to alleviate apoptosis as a heat stress therapeutic drug has not been explored.

At present, heat stress is still a prominent problem that threatens human and animal health in tropical and subtropical areas. The use of bioactive products to alleviate stress injury is highly practical. Therefore, this study aimed to establish a heat shock model using IPEC-J2 cells and a heat stress model in mice to investigate the molecular mechanism through which BTL-I intervenes in intestinal epithelial cell apoptosis.

## 2. Results

### 2.1. Effect of BTL-I on the Expression of Related Factors in Heat-Shocked IPEC-J2 Cells

**Figure 1:**
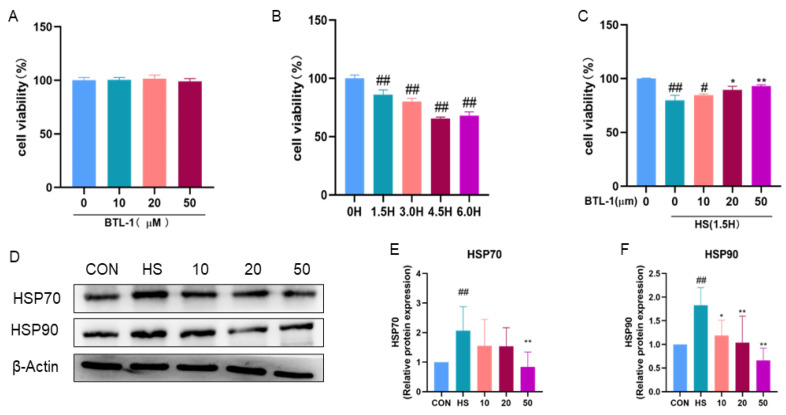
Effect of BTL-I on the expression of related factors in heat-shocked IPEC-J2 cells. IPEC-J2 cells were treated with different concentrations of BTL-I (10, 20, or 50 μM) for 24 h and then coincubated with the heat shock (HS) group in a 5% CO_2_ incubator at 42 °C for 1.5 h. Cell viability was determined via a CCK8 assay. (**A**) Effects of BTL-I on the viability of IPEC-J2 cells; (**B**) effects of heat shock treatment on the viability of IPEC-J2 cells; (**C**) effects of BTL-I and heat shock cotreatment on IPEC-J2 cells; and (**D**–**F**) the expression levels of the heat shock proteins HSP70 and HSP90 were detected via Western blotting. The results are expressed as the means ± SEMs. ^#^ *p* < 0.05, ^##^ *p* < 0.01 compared with the control group; * *p* < 0.05, ** *p* < 0.01 compared with the HS group.

The cytotoxicity of BTL-I on IPEC-J2 cells was evaluated via the CCK8 assay. After IPEC-J2 cells were exposed to three different concentrations of BTL-I for 24 h, the cell proliferation rate was unaffected (Figure 1A). However, as the duration of the heat shock treatment increased (1.5 h, 3.0 h, 4.5 h, and 6.0 h), there was a notable and significant decrease in the cell proliferation rate (*p* < 0.01). Since the cell proliferation rate was still greater than 80% after 1.5 h of heat shock treatment, subsequent assessments were conducted at this time point (Figure 1B).

Moreover, after pretreatment with BTL-I, the IPEC-J2 cells exhibited a dose-dependent increase in the cell proliferation rate following heat shock (Figure 1C). Additionally, Western blot analysis revealed that the expression levels of HSP70 and HSP90 significantly increased (*p* < 0.01) following heat shock treatment. Conversely, the expression levels of these proteins were significantly reduced (*p* < 0.05) after BTL-I pretreatment (Figure 1D–F).

### 2.2. Effects of BTL-I on the Apoptosis of Heat-Shocked IPEC-J2 Cells

**Figure 2:**
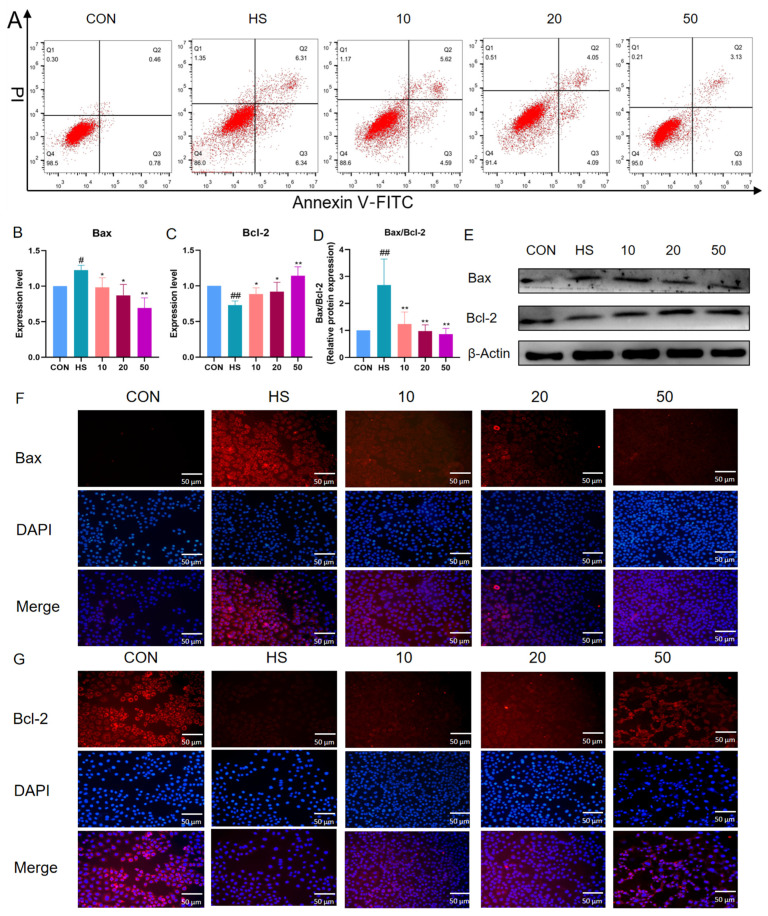
Effects of BTL-I on IPEC-J2 cell apoptosis after heat shock. (**A**) Flow cytometry analysis of the cell apoptosis rate; (**B**,**C**) qPCR analysis of *Bax* and *Bcl-2*; (**D**,**E**) Western blotting analysis of Bax and Bcl-2; and (**F**,**G**) IF analysis of Bax and Bcl-2; the red fluorescence represents Bcl-2 and Bax protein expression, and the blue fluorescence corresponds to the cell nuclei; the results are expressed as the means ± SEMs. ^#^ *p* < 0.05, ^##^ *p* < 0.01 compared with the control group; * *p* < 0.05, ** *p* < 0.01 compared with the HS group.

We assessed the impact of BTL-I on IPEC-J2 cell apoptosis. The findings indicated that the apoptosis rate of the IPEC-J2 cells increased following heat shock treatment compared with that in the CON group. However, after pretreatment with BTL-I, the percentage of IPEC-J2 cells that underwent apoptosis decreased (Figure 2A). Further analysis of apoptosis-related proteins revealed that the mRNA expression of *Bax* significantly increased (*p* < 0.05) after heat shock, but decreased following BTL-I pretreatment (Figure 2B). The mRNA expression of *Bcl-2* had the opposite effect (*p* < 0.05) (Figure 2C). BTL-I pretreatment led to a significant decrease in the Bax/Bcl-2 protein ratio, as determined by Western blotting (*p* < 0.01) (Figure 2D,E). Additionally, the immunofluorescence results demonstrated that the fluorescence intensity of Bcl-2 was more pronounced in the IPEC-J2 cells treated with BTL-I after heat shock, whereas the fluorescence intensity of Bax decreased upon BTL-I addition (Figure 2F,G).

### 2.3. Effect of BTL-I on the ROS/PERK/CHOP Signaling Pathway in IPEC-J2 Cells Subjected to Heat Shock

**Figure 3:**
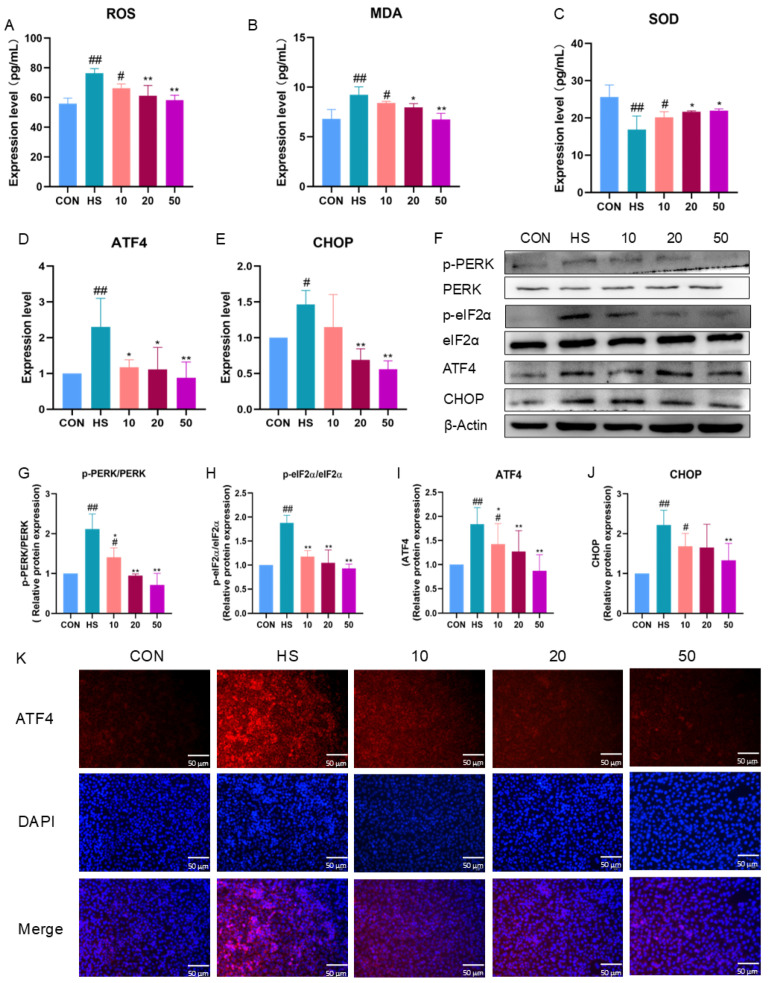
Effect of BTL-I on the ROS/PERK/CHOP signaling pathway in IPEC-J2 cells subjected to heat shock. (**A**–**C**) ELISA analysis of the levels of the oxidative markers ROS, MDA, and SOD; (**D**,**E**) qPCR analysis of the levels of the PERK/CHOP signaling pathway markers *ATF4* and *CHOP*; (**F**–**J**) Western blotting analysis of the levels of the PERK/CHOP signaling pathway markers p-PERK, PERK, p-eIF2α, eIF2α, ATF4, and CHOP; and (**K**) IF analysis of the levels of the PERK/CHOP signaling pathway marker ATF4. The results are expressed as the means ± SEMs. ^#^ *p* < 0.05, ^##^ *p* < 0.01 compared with the control group; * *p* < 0.05, ** *p* < 0.01 compared with the HS group.

To investigate the mechanism underlying heat-shock-induced cell apoptosis, we examined the response of the ROS/PERK/CHOP signaling pathway, and the results are illustrated in Figure 3. Following heat shock (HS), there was a significant increase in the ROS and MDA levels in the IPEC-J2 cells (*p* < 0.01), as well as a decrease in SOD expression (*p* < 0.01) (Figure 3A–C). However, the addition of BTL-I effectively reversed the increases in ROS and MDA and the decrease in SOD (*p* < 0.05).

Furthermore, after heat shock treatment, the mRNA expression of *ATF4* and *CHOP* was significantly increased (*p* < 0.05). However, the expression of these genes was significantly downregulated after pretreatment with BTL-I (*p* < 0.05) (Figure 3D,E). Western blotting revealed significant increases in p-PERK/PERK and p-eIF2α/eIF2α expression (*p* < 0.01) after heat shock, which was consistent with the changes observed in ATF4 and CHOP expression (*p* < 0.01). However, their expression levels decreased following BTL-I pretreatment (Figure 3F–J). The immunofluorescence results indicated that the fluorescence intensity of ATF4 increased upon heat shock treatment, whereas BTL-I pretreatment led to a reduction in its expression (Figure 3K).

### 2.4. Effect of ROS Scavengers on the PERK/CHOP Signaling Pathway in Heat-Shocked IPEC-J2 Cells

**Figure 4:**
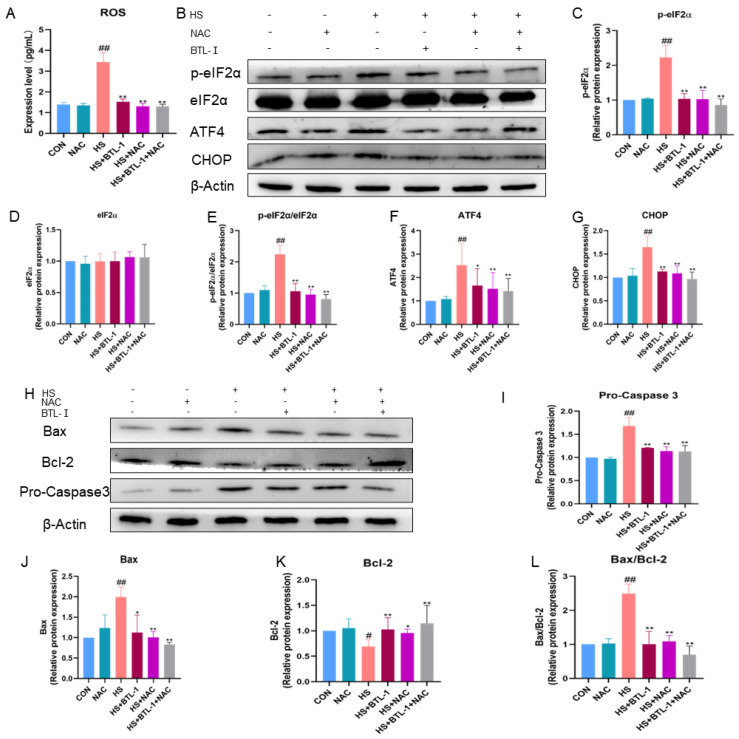
Effects of ROS scavengers on the PERK/CHOP signaling pathway and apoptosis in heat-shocked IPEC-J2 cells. IPEC-J2 cells were treated with BTL-I (50 μM) or NAC (1 mM) for 24 h or 3 h, respectively, and then placed together with the heat shock (HS) group in a cell incubator at 42 °C and 5% CO_2_ for 1.5 h. (**A**) ELISA analysis of the oxidative marker ROS. (**B**–**G**) Western blot analysis of the expression of the PERK/CHOP signaling pathway markers p-PERK, PERK, p-eIF2α, eIF2α, ATF4, and CHOP. (**H**–**L**) Western blot analysis of the expression of the apoptosis markers Bax/Bcl-2 and procaspase 3. The results are expressed as the means ± SEMs. ^#^ *p* < 0.05, ^##^ *p* < 0.01 compared with the control group; * *p* < 0.05, ** *p* < 0.01 compared with the HS group.

To further investigate whether BTL-I inhibits apoptosis through endoplasmic reticulum stress (ERS), we examined the expression of PERK/CHOP-signaling-pathway-related proteins, as shown in Figure 4. The addition of the ROS scavenger NAC effectively reversed the heat-shock-induced increase in ROS accumulation (*p* < 0.01) (Figure 4A). Following heat shock treatment, the protein expressions of p-eIF2α/eIF2α, ATF4, and CHOP significantly increased (*p* < 0.01). However, the expressions of these proteins were notably inhibited after pretreatment with BTL-I or NAC (*p* < 0.05) (Figure 4B–G).

Moreover, compared with those in the CON group, the protein expression levels of Bax/Bcl-2 in the heat shock group were significantly greater (*p* < 0.01); however, pretreatment with 1 mM NAC significantly downregulated the expression of these genes (*p* < 0.01) (Figure 4H–L). These results suggest that BTL-I may exert its antiapoptotic effects through the inhibition of the ERS pathway and that the ROS scavenger NAC contributes to this protective effect.

**Figure 5:**
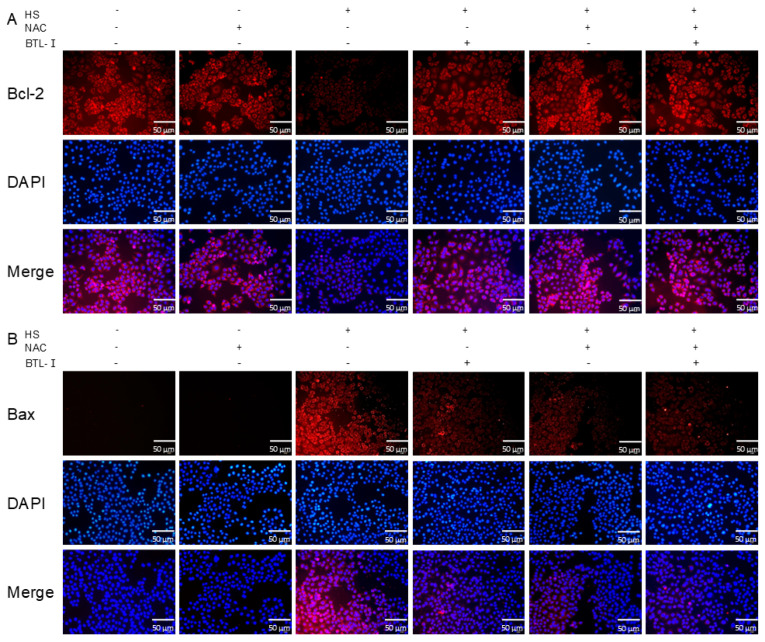
Effect of ROS scavengers on apoptosis in heat-shocked IPEC-J2 cells. (**A**,**B**) IF analysis of the apoptosis markers Bax and Bcl-2.

The immunofluorescence results demonstrated that, upon the addition of NAC, the fluorescence intensity of Bcl-2 was more pronounced than that in the IPEC-J2 cells treated with heat shock alone. Conversely, the fluorescence intensity of Bax decreased after the addition of NAC (Figure 5A,B).

### 2.5. Effect of an ER Stress Inhibitor on the Apoptosis of Heat-Shocked IPEC-J2 Cells

**Figure 6:**
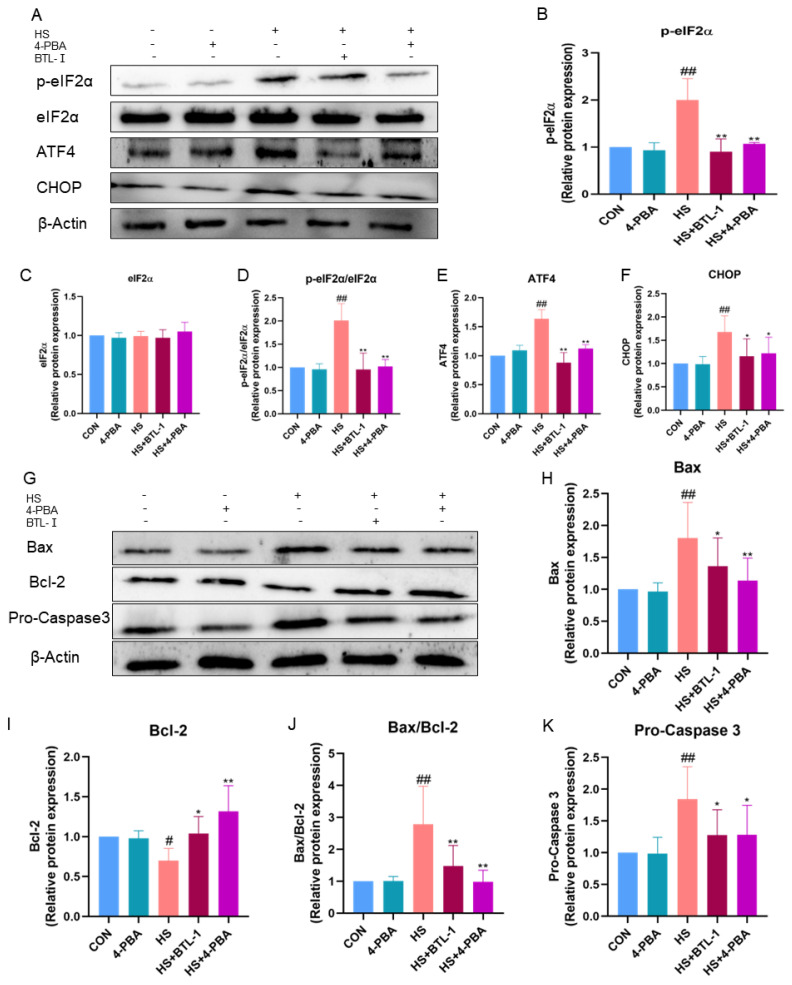
Effect of an ER stress inhibitor on the apoptosis of heat-shocked IPEC-J2 cells. IPEC-J2 cells were treated with BTL-I (50 μM) or 4-PBA (1 mM) for 24 h or 3 h, respectively, and then placed together with the heat shock (HS) group in a cell incubator at 42 °C and 5% CO_2_ for 1.5 h. (**A**–**F**) Western blot analysis of the expression of the PERK/CHOP signaling pathway markers p-PERK, PERK, p-eIF2α, eIF2α, ATF4, and CHOP. (**G**–**K**) Western blot analysis of the expression of the apoptosis markers Bax/Bcl-2 and procaspase 3. The results are expressed as the means ± SEMs. ^#^ *p* < 0.05, ^##^ *p* < 0.01 compared with the control group; * *p* < 0.05, ** *p* < 0.01 compared with the HS group.

To investigate the role of the PERK/CHOP signaling pathway, we pretreated cells with the ER stress inhibitor 4-PBA, and the results are shown in Figure 6. Compared with those in the heat shock group, the protein expression levels of p-eIF2α/eIF2α, ATF4, and CHOP were significantly lower after pretreatment with 1 mM 4-PBA (*p* < 0.05) (Figure 6A–F).

Furthermore, following pretreatment with 1 mM 4-PBA, the protein expressions of Bax/Bcl-2 and pro-caspase 3 were significantly lower than those in the HS group (*p* < 0.05) (Figure 6G–K). These findings indicate the potential involvement of the PERK/CHOP signaling pathway in these effects, and the ER stress inhibitor 4-PBA appears to play a role in mitigating apoptosis.

**Figure 7:**
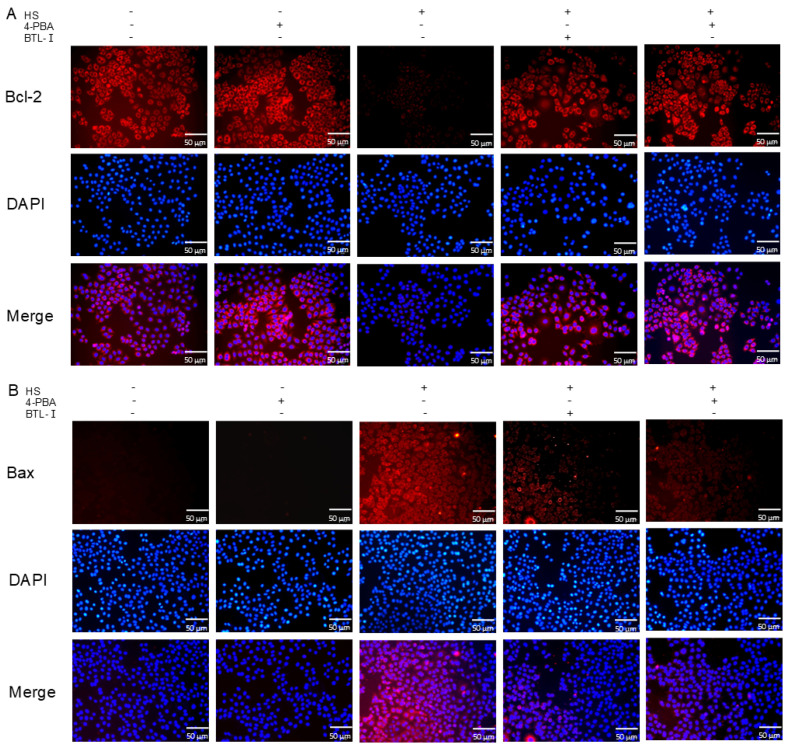
Effect of an endoplasmic reticulum inhibitor on the apoptosis of heat-shocked IPEC-J2 cells. (**A**,**B**) IF analysis of the apoptosis markers Bax and Bcl-2.

The immunofluorescence results revealed that, upon the addition of 4-PBA, the fluorescence intensity of Bcl-2 was more pronounced than that in the IPEC-J2 cells treated with heat shock alone. Conversely, the fluorescence intensity of Bax decreased after the addition of 4-PBA (Figure 7A,B).

### 2.6. Protective Effect of BTL-I on Heat-Stressed Mice

**Figure 8:**
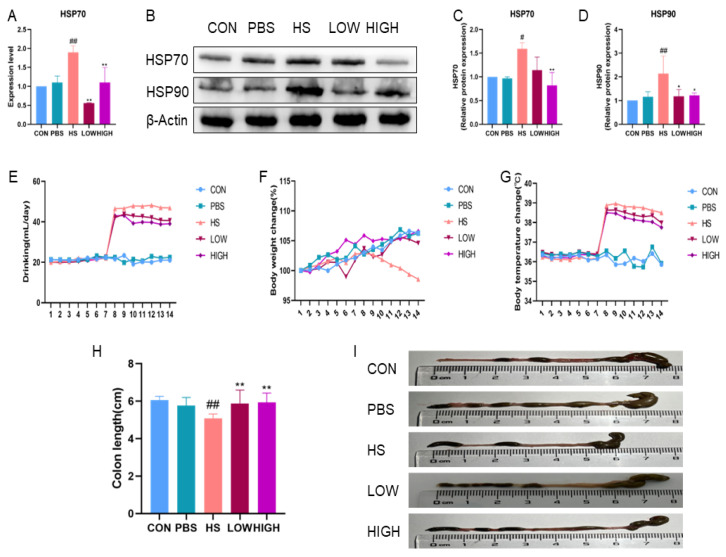
Protective effect of BTL-I on heat-stressed mice. The normal control group (CON) was exposed to 24 ± 1 °C and treated with PBS (0.2 mL), the heat stress group (HS) was subjected to 40 ± 1 °C for 4 h per day, the HS group had a low oral BTL-I concentration (1 mg/kg) (LOW), and the HS group had a high oral BTL-I concentration (5 mg/kg) (HIGH). Clinical changes were recorded, and colon tissues were collected. (**A**–**D**) qPCR and Western blotting analysis of the effects of BTL-I on HSP70 and HSP90 proteins; (**E**) water intake of the mice during the test; (**F**) body weight changes in the mice during the test; (**G**) body temperature changes; and (**H**,**I**) colon length of the mice during the test; the results are expressed as the means ± SEMs. ^#^ *p* < 0.05, ^##^ *p* < 0.01 compared with the control group; * *p* < 0.05, ** *p* < 0.01 compared with the HS group.

We examined the impact of BTL-I on heat-stressed mice, and the results are presented in Figure 8. The expression of *HSP70* mRNA in the HS group was significantly upregulated (*p* < 0.01). Conversely, in the BTL-I group, the expression of *HSP70* mRNA was significantly downregulated (*p* < 0.01), with the most notable effect observed at a BTL-I dose of 5 mg/kg (Figure 8A). Furthermore, Western blotting revealed significant increases in the expressions of HSP70 and HSP90 after heat stress (*p* < 0.05). However, following BTL-I pretreatment, the expression levels of these genes decreased significantly (*p* < 0.05) (Figure 8B–D). As the duration of heat stress increased, the weight of the mice gradually decreased, whereas their water intake and body temperature increased (Figure 8E–G). However, after the oral administration of BTL-I, weight loss was mitigated, and water intake and body temperature gradually returned to normal levels. Additionally, the colon length of the mice decreased after heat stress, but this change was reversed after the oral administration of BTL-I (Figure 8H–I). These results suggest that BTL-I has a beneficial effect on mitigating the adverse impacts of heat stress in mice.

### 2.7. Effects of BTL-I on Intestinal Cell Apoptosis in Heat-Stressed Mice

**Figure 9:**
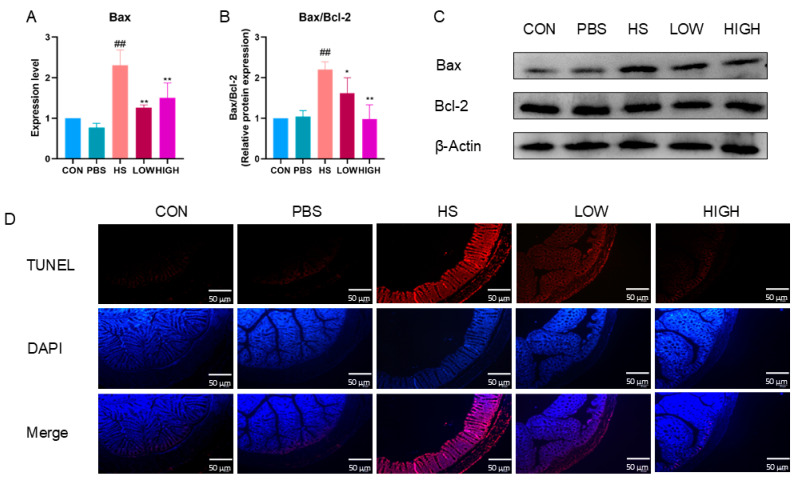
Effects of BTL-I on apoptosis in heat-stressed mice. (**A**) The effects of BTL-I on the expression of the apoptotic marker *Bax* were determined by qPCR. (**B**–**C**) The effects of BTL-I on the expression of the apoptosis markers Bax, Bcl-2, and Pro-Caspase 3 were determined by Western blotting. (**D**) Immunofluorescence staining for apoptosis was performed via the TUNEL assay. The results are expressed as the means ± SEMs. ^##^ *p* < 0.01 compared with the control group; * *p* < 0.05, ** *p* < 0.01 compared with the HS group.

As depicted in Figure 9A, the mRNA expression level of *Bax* was significantly increased after heat stress (HS) (*p* < 0.01). However, following BTL-I administration, the mRNA level of *Bax* was significantly decreased (*p* < 0.01). Moreover, the protein expression of Bax/Bcl-2 significantly increased after heat stress (*p* < 0.05). Nonetheless, BTL-I administration notably reversed these changes (*p* < 0.05) (Figure 9B,C). Furthermore, the TUNEL results indicated that intestinal cell apoptosis was greater in the BTL-I-treated mice than in the HS-treated mice (Figure 9D). These findings suggest that BTL-I may protect against heat-stress-induced intestinal cell apoptosis.

### 2.8. Effect of BTL-I on the ROS/PERK/CHOP Signaling Pathway in Heat-Stressed Mice

**Figure 10:**
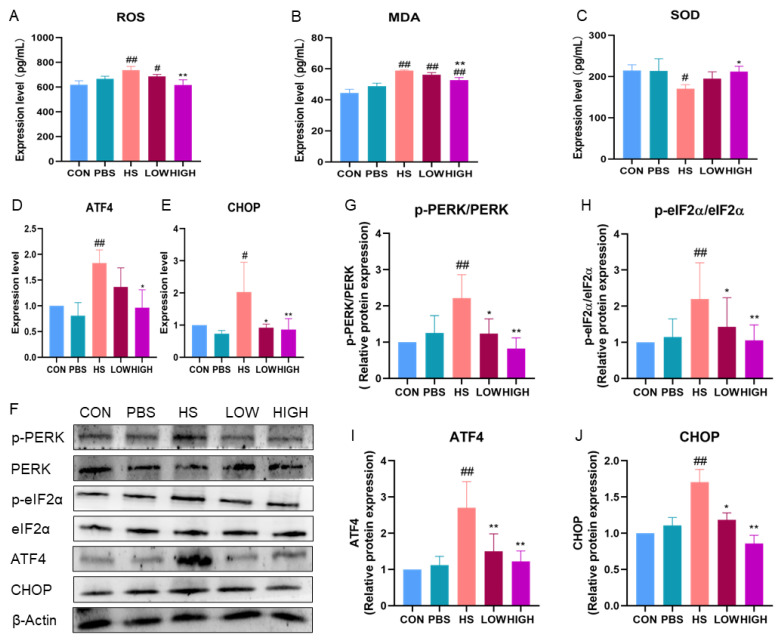
Effects of BTL-I on the ROS/PERK/CHOP signaling pathway in heat-stressed mice. (**A**–**C**) The effects of BTL-I on the expression of oxidative cytokines were determined by ELISA; (**D**,**E**) qPCR analysis of the expression of the PERK/CHOP signaling pathway markers *ATF4* and *CHOP*; and (**F**–**J**) Western blotting analysis of the expression of the PERK/CHOP signaling pathway markers p-PERK, PERK, p-eIF2α, eIF2α, ATF4, and CHOP; the results are expressed as the means ± SEMs. ^#^ *p* < 0.05, ^##^ *p* < 0.01 compared with the control group; * *p* < 0.05, ** *p* < 0.01 compared with the HS group.

We examined the expression of oxidative-stress-related molecules in mouse serum. The results revealed that the levels of ROS and MDA in the serum of HS mice were significantly increased (*p* < 0.05) (Figure 10A–C). However, BTL-I administration effectively inhibited the increases in the ROS and MDA levels (*p* < 0.01). Additionally, HS led to a reduction in the content of the antioxidant factor SOD in the serum (*p* < 0.05), and BTL-I administration significantly alleviated this decrease caused by HS (*p* < 0.05).

Furthermore, we explored the mechanism by which BTL-I inhibited endoplasmic reticulum stress (ERS) in HS mice by examining the key proteins of the PERK/CHOP signaling pathway. HS increased the expressions of the *ATF4* and *CHOP* mRNAs (*p* < 0.05). Additionally, the expression levels of p-PERK/PERK, p-eIF2α/eIF2α, ATF4, and CHOP were significantly increased (*p* < 0.01). However, after BTL-I administration, these changes were abolished (*p* < 0.05) (Figure 10D–J). These findings suggest that BTL-I may ameliorate ERS by modulating the PERK/CHOP signaling pathway in HS mice.

## 3. Discussion

Heat stress is still a prominent threat to animal husbandry in tropical and subtropical areas. Studying and developing natural active products to alleviate stress damage is highly practical. BTL-I has good anti-inflammatory, antioxidant, and intestinal balance effects, but whether BTL-I can alleviate heat stress damage and develop as a functional feed additive for livestock and poultry remains unclear. In this study, we investigated the protective mechanism of BTL-I against heat-stress-induced apoptosis in IPEC-J2 cells and intestinal cells in mice. Our results demonstrated that heat stress led to an increase in heat shock protein levels, which were effectively inhibited by BTL-I. Both in vivo and in vitro experiments revealed that heat stress increased the rate of intestinal epithelial cell apoptosis and increased the expression of apoptosis-related proteins. However, BTL-I treatment improved these effects, primarily through the regulation of the ROS/PERK/CHOP signaling pathway. These findings suggest that BTL-I has the potential to prevent heat-stress-induced intestinal cell apoptosis.

Apoptosis is a process of programmed cell death observed in pathological and specific physiological states and is characterized by active, orderly, and gene-regulated physiological changes that can alter cell shape and function [18,19]. Studies have indicated that heat stress can lead to the accumulation of ROS and mitochondrial dysfunction, triggering cell apoptosis [20]. Additionally, heat stress can induce apoptosis in mammary epithelial cells by downregulating ROS-independent pathways [21]. Acute heat stress has been associated with p53-mediated mitochondrial apoptosis in liver injury [22]. Moreover, Cui reported that acute heat stress activates the unfolded protein response (UPR), leading to endoplasmic reticulum stress (ERS) and subsequent apoptosis [23]. High temperatures can induce cell apoptosis or necrosis, and the degree of apoptosis or necrosis becomes more evident as the temperature increases [24,25].

To investigate the effects of BTL-I on intestinal cell apoptosis, the IPEC-J2 cell heat shock model and C57BL/6J mouse heat stress model were used. In our study, we observed increased apoptosis rates and upregulated expressions of Bax/Bcl-2 and Pro-Caspase 3 in IPEC-J2 cells and mice exposed to heat stress. However, pretreatment with BTL-I resulted in a reduction in the apoptosis rate and the downregulation of Bax/Bcl-2 and Pro-Caspase 3 expression. These findings highlight the potential of BTL-I as a preventive agent against heat-stress-induced intestinal cell apoptosis.

The endoplasmic reticulum (ER) plays a vital role in protein folding and maturation. ER stress, caused by unfolded or misfolded proteins, can lead to cell apoptosis. Many factors cause ER proteins to misfold or unfold, such as humidity, Ca^2+^ level disturbance, oxidative stress, nutritional deficiencies, and ischemia [26,27,28]. Endoplasmic reticulum stress can damage the morphological structure and physiological functions of the endoplasmic reticulum, resulting in many unfolded or misfolded proteins [29,30]. At this time, cells undergo a highly conserved stress-adaptive response (UPR), which mainly reduces the synthesis of new proteins by shutting down protein translation, inducing ER chaperones that activate protein folding, and ubiquitinating misfolded proteins/unfolded proteins, thereby relieving ER stress, protecting the ER, and reducing damage [31,32,33]. However, long-term stress stimulation makes the UPR insufficient to relieve endoplasmic reticulum stress and eventually leads to cell apoptosis, resulting in the occurrence of disease. For example, in the treatment of prostate cancer, resveratrol triggers ER stress by depleting ER Ca^2+^ and inducing autophagy-mediated apoptosis [34,35]. Similarly, cucurbitacin I induces cell death by activating the PERK and IRE1α signaling pathways while increasing Bax and caspase-12-dependent ER stress to induce cell apoptosis [36]. Moreover, heat stress (HS) can induce ER stress, activate the p-eIF2α/CHOP signaling pathway, trigger the apoptosis of intestinal epithelial cells, and compromise the integrity of the intestinal epithelial barrier [37]. The in vitro exposure of intestinal epithelial cells to high temperatures (41 °C) decreases cell viability, induces ER stress, and promotes cell apoptosis, ultimately disrupting barrier function [38]. Similarly, our study revealed that heat stress can induce ER stress and upregulate the PERK, eIF2α, ATF4, and CHOP proteins in IPEC-J2 and mouse cells.

Free radicals, including reactive oxygen species (ROS) and reactive nitrogen species (RNS), possess strong oxidation abilities and play significant roles in cell proliferation, differentiation, growth, and death. They are also involved in various cellular processes, such as the immune system, redox balance, and the activation of signaling pathways, including the PI3K/Akt, MAPK, eIF2α/ATF4/CHOP, and Nrf2/Keap1 pathways [39,40,41,42,43,44]. ROS, a byproduct of the electron respiratory transfer chain of mitochondria, are associated with inflammation, ER stress, and metabolic diseases due to the disruption of ROS levels [31,45,46,47,48]. MCL can induce apoptosis by targeting TrxR, triggering ROS-mediated ERS, and then inducing ICD in HCC cells [49]. Brd4 inhibition prevents FoxO4-dependent ROS production through the PI3K/AKT pathway, thereby blocking renal cell apoptosis and ERS protein expression [50]. Zinc-based ICD inducers induce mitochondrial dysfunction through ROS induction via ERS [51]. On the basis of previous reports, we hypothesized that heat-stress-induced apoptosis in IPEC-J2 and mouse intestinal epithelial cells is also regulated by the ROS/PERK/CHOP signaling pathway. To validate this hypothesis, we examined the ROS levels and observed that HS led to ROS accumulation in IPEC-J2 and mouse cells. These findings suggest that the ROS/PERK/CHOP signaling pathway mediates heat-stress-induced apoptosis.

N-acetylcysteine (NAC) scavenges reactive oxygen species (ROS) and effectively eliminates accumulated ROS [52]. In previous studies, NAC was shown to mitigate intestinal epithelial cell apoptosis by inhibiting the heat-labile enterotoxin-induced activation of the PERK/CHOP pathway [53]. Additionally, in an ischemia–reperfusion-induced acute kidney injury model, the addition of NAC effectively suppressed ROS-mediated ER stress and cell apoptosis signaling pathways [54]. In addition, TiO_2_-NP-induced ER stress was significantly reduced in HT22 cells treated with NAC, as were GRP78 and caspase 12 expression [55]. In our study, the addition of NAC effectively reduced the expressions of p-PERK, p-eIF2α, ATF4, and CHOP, thereby reducing the occurrence of apoptosis. On the other hand, 4-phenyl butyric acid (4-PBA) acts as an ER stress inhibitor, clearing misfolded and unfolded proteins and inhibiting ER stress [56]. In various studies, 4-PBA has been demonstrated to inhibit ER stress and prevent acute lung injury induced by a hyperoxygen environment by upregulating Claudin 4 expression [57]. Moreover, when ER stress is inhibited, IFN-γ-mediated apoptosis is also reduced, resulting in decreased unfolded and misfolded protein responses [58]. Our study also revealed that 4-PBA effectively inhibited the expressions of p-PERK, p-eIF2α, ATF4, and CHOP and reduced the expressions of the apoptosis-related proteins Pro-Caspase 3 and Bax/Bcl-2. The results obtained with NAC and 4-PBA were consistent with those obtained with BTL-I, indicating that BTL-I mitigates heat-stress-induced apoptosis by modulating the ROS/PERK/CHOP signaling pathway. These findings further support the potential of BTL-I as a preventive measure against heat-stress-induced intestinal cell apoptosis.

The Keap1-Nrf2/ARE signaling pathway is one of the main pathways of anti-oxidative stress in vivo [59]. It can resist oxidative stress caused by various stimulating factors inside and outside the body, and improve the anti-oxidative damage and repair function of cells. It is the central defense mechanism of the system against oxidative stress. Nrf2 is a key transcription factor and the main regulator of the cellular oxidative stress response, which can induce the activation of the Nrf2-mediated expression of antioxidant enzymes, detoxification enzymes, and downstream protein molecules, such as NQO1, SOD, CAT, HO-1, and GPX [60,61]. At the same time, under environmental pressure or stimuli, ROS levels will increase dramatically, causing serious harm to cells, leading to cell function damage and even death. When a large amount of ROS accumulates in cells, Keap1 dissociates from Nrf2 and induces Nrf2 translocation to the nucleus to bind to antioxidant elements, promoting the transcription of antioxidant-related genes [62,63]. So, Nrf2 activation can effectively remove ROS accumulation and reduce oxidative stress damage. Based on the above studies, we will continue to explore whether BTL-I can alleviate apoptosis by activating Nrf2 to reduce ROS in IPEC-J2 cells in the future.

## 4. Materials and Methods

### 4.1. Chemicals and Reagents

BTL-I was purchased from Bioviotica (San Diego, CA, USA). Its chemical formula was C24H24O7. The purity of BTL-I was 99.9%. A Cell Counting Kit-8 (CCK8) was purchased from APExBIO Technology (Houston, TX, USA). Commercial-specific complete Dulbecco’s modified Eagle’s medium/nutrient mixture F-12 (DMEM/F12), penicillin, and streptomycin were acquired from Gibco (Carlsbad, CA, USA). An enhanced chemiluminescence (ECL) kit was obtained from Tanon (Shanghai, China). In addition, 40,6-diamidino-2-phenylindole (DAPI) was purchased from Beyotime (Shanghai, China). The primary and secondary antibodies used for Western blotting were purchased from Cell Signaling Technology (Danvers, MA, USA), Abcam (Cambridge, MA, USA) and Proteintech (Wuhan, China). Biotin-conjugated anti-rabbit IgG antibody and DyLight 594-conjugated avidin were purchased from Jackson ImmunoResearch (Cambridge, MA, USA).

### 4.2. Cell Culture and Viability Assay

IPEC-J2 cells obtained from Guoqiang Zhu at Yangzhou University (Yangzhou, China) and were cultured in DMEM/F12 supplemented with 10% FBS and 1% penicillin/streptomycin at 37 °C. After reaching 80% confluence, the following two sets of cells were prepared: one group was subjected to heat shock treatment, and the other group was subjected to BTL-I pretreatment. The heat shock treatment involved placing the cells in a cell incubator at 42 °C with 5% CO_2_ for 1.5 h.

To assess IPEC-J2 cell viability, a CCK8 assay was performed. Initially, the cells were seeded at a density of 1 × 10^4^ cells/mL in 96-well plates and treated with varying concentrations of BTL-I (10, 20, and 50 µM) for 24 h. The experimental concentration was set according to previously published methods [64]. Afterward, 10 µL of CCK-8 solution was added, and the cells were further incubated for 2 h. A microplate reader (BioTek, Winooski, VT, USA) was used to measure cell viability by measuring the absorbance at 450 nm.

### 4.3. Induction of Heat Stress in Mice

Male C57BL/6J mice aged 6–8 weeks and weighing 19–21 g were procured from a specific pathogen-free (SPF) laboratory located in Guangzhou, China. The mice were housed in a controlled environment with a temperature of 26 ± 1 °C and a 12 h light/dark cycle. All animal protocols adhered to the guidelines of the IACUC-Guangdong Ocean University and were granted ethics approval (number 2022-scuec-021). The animal experiments strictly followed the principles outlined in the National Research Council’s Guide for the Care and Use of Laboratory Animals (National Institutes of Health, Bethesda, MD, USA).

**Figure 11:**
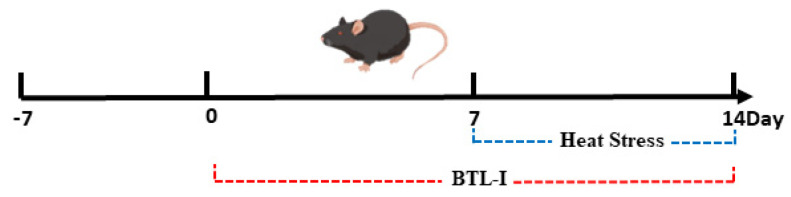
Mouse experiment.

Before the experiments commenced, the mice were acclimatized for one week, during which they had unrestricted access to food and water. The mice, exposed to a relative humidity (RH) of 65–85%, were randomly divided into the following five groups (*n* = 6): the normal control group (−HS) exposed to 24 ± 1 °C, the normal control group treated with PBS (0.2 mL), the heat stress group (+HS) subjected to 40 ± 1 °C for 4 h per day, the HS group with a low oral BTL-I concentration (1 mg/kg) (L-BTL-I), and the HS group with a high oral BTL-I concentration (5 mg/kg) (H-BTL-I). The experimental concentrations were set according to previously published methods [64]. This treatment regimen was continued for 14 days (Figure 11). The mice were subsequently euthanized, and their colons were collected for further research.

### 4.4. Detection of Oxidative Stress Markers

To examine the inhibitory effect of BTL-I on oxidative cytokines, IPEC-J2 cells were initially seeded at a density of 5 × 10^6^ cells/well in 6-well plates. The cells were then pretreated with different concentrations of BTL-I (10, 20, and 50 µM) for 24 h, followed by exposure to heat stress (HS) for 1.5 h. Subsequently, the cells were disrupted via an ultrasonic cell disruptor (Ningbo Shuangjia Instrument Co., Ltd., Ningbo, China) and centrifuged at 3000 rpm for 10 min. The resulting serum was collected and subjected to further centrifugation at 3000 rpm for an additional 10 min. The expression levels of ROS, MDA, and SOD (Jiangsu Meimian Industrial Co., Ltd., Nanjing, China) were detected according to the instructions of the ELISA kit according to the manufacturer’s instructions.

### 4.5. Western Blotting Analysis

Protein levels were assessed via a BCA reagent after the homogenization of IPEC-J2 cells and colonic tissues (CWBIO, Beijing, China). To analyze the samples, sodium dodecyl sulfate–polyacrylamide gel electrophoresis (SDS–PAGE) was employed, followed by the transfer of the separated proteins to polyvinylidene fluoride (PVDF) membranes. The PVDF membranes were subsequently blocked for 30 min with QuickBlockTM Blocking Buffer (Beyotime, Shanghai, China). Following blocking, the membranes were incubated with primary antibodies at a 1:1000 dilution. The primary antibodies used were against HSP70 (Proteintech, 10995-1-AP), HSP90 (Abcam, ab59459), PERK (CST, 5683s), eIF2α (CST, 3179s), p-PERK (CST, 5324s), p-eIF2α (CST, 3398s), ATF4 (CST, 11815s), CHOP (Abcam, ab11419), Bax (Proteintech, 60267-1-lg), Bcl-2 (Proteintech, 60267-1-lg), and β-actin (TransGen Biotech, Lot# R10602). For the next step, secondary antibodies, namely, goat anti-rabbit and anti-mouse IgG conjugated with horseradish peroxidase (HRP), were used at a 1:1000 dilution.

The Western blot normalization method is shown below. The first step was to quantify the target protein and housekeeping protein in each lane. Next, the reference lane on the blot was selected. The reference lane was the CON group lane. Then, the normalization factor was determined by dividing the signal of the reference channel by the signals of the other channels, the normalization factor of each channel was obtained, and the signal of the target protein was multiplied by the normalization factor.

### 4.6. Total RNA Extraction and qPCR

RNA extraction was performed via TRIzol, and the isolated RNA was subsequently reverse-transcribed into cDNA. To assess the gene expression levels, SYBR fluorescence quantitative PCR was used, and the obtained data were normalized via the 2^−ΔΔCT^ method, with β-actin serving as the reference gene. The specific primer sequences utilized in the PCR analysis can be found in Table 1.

### 4.7. Immunofluorescence

IPEC-J2 cells at 50% confluency were treated with BTL-I in a 24-well plate. The cells were subsequently gently washed, fixed, and blocked. Next, the cells were incubated with anti-Bcl-2 (1:100; Abcam, Cambridge, MA, USA), anti-ATF4, and anti-Bax antibodies (1:100; Cell Signaling Technology, Danvers, MA, USA) overnight in the dark at 4 °C. After thorough washing with PBS, the cells were incubated with the appropriate secondary antibody conjugated with DyLight 594 fluorescent probes in the dark for 2 h. Subsequently, the cells were washed again with PBS and stained with a fluorescently labeled protein (DyLight 594-conjugated avidin) for 1 h. Finally, the cells were observed under a fluorescence microscope (Olympus, Tokyo, Japan) with DAPI-stained nuclei.

### 4.8. Flow Cytometry

The apoptosis analysis was conducted via the Annexin V-FITC/PI Apoptosis Detection Kit from Yeasen Biotechnology (Shanghai, China) following the provided instructions. Cell samples were collected and subjected to flow cytometry to determine the percentage of apoptotic cells. The percentage of apoptotic cells was calculated as the sum of the percentages in the first and fourth quadrants of a two-dimensional scatter plot.

### 4.9. H&E Staining

Colon tissue obtained from the mice was preserved in 4% phosphate-buffered paraformaldehyde. Afterward, the tissues were embedded in paraffin, and thin sections were prepared. These sections were then stained with hematoxylin–eosin (H&E), after which, pathological changes were observed via optical microscopy (Olympus, Tokyo, Japan).

### 4.10. Terminal Deoxynucleotidyl Transferase-Mediated dUTP-Biotin Nick End Labeling (TUNEL) Assay

Following the guidelines of Yeasen Biotechnology (Shanghai, China), the paraffin sections were dewaxed. Proteinase K treatment was conducted for 20 min, followed by incubation with equilibration buffer for 10 min. The sections were then subjected to TdT solution and incubated for 30 min at 37 °C. Finally, DAPI was added to the sealing solution, which enabled observation and imaging under a fluorescence microscope (Olympus, Tokyo, Japan).

### 4.11. Statistical Analyses

One-way analysis of variance, followed by Tukey’s multiple comparisons test (LSD), was performed via IBM SPSS 19.0 (SPSS, Chicago, IL, USA). The results are expressed as the mean ± SEM. *p* < 0.05 was considered to indicate statistical significance. Graphs were created via GraphPad Prism 8.0 software (San Diego, CA, USA).

## 5. Conclusions

In conclusion, our study demonstrated that BTL-I exerts a beneficial effect on heat-stress-induced apoptosis by regulating the ROS-mediated PERK/CHOP signaling pathway, effectively inhibiting apoptosis. The positive effects of BTL-I in the heat stress model suggest its potential as a novel marine drug for preventing heat stress. These findings provide a solid theoretical basis for the defense against and treatment of heat stress, opening up new possibilities for managing this condition. Owing to the complex preparation process and high cost of BTL-I, it is not suitable for livestock breeding at present. With continuous optimization of the preparation process, BTL-I is expected to be used as a feed additive in pig production.

## Figures and Tables

**Table 1:** Mouse primer sequences.

Gene Name	Sequence (5′-3′)
*m-ATF4*	F: TCTGCCTTCTCCAGGTGGTTCC
	R: GCTGCTGTCTTGTTTTGCTCCATC
*m-CHOP*	F: CTACTCTTGACCCTGCGTCCCTAG
	R: TCTTCCTTGCTCTTCCTCCTCTTCC
*m-HSP70*	F: GGTGCTGACGAAGATGAAGGAGATC
	R: CTGCCGCTGAGAGTCGTTGAAG
*m-Bcl-2*	F: GATGACTTCTCTCGTCGCTAC
	R: GAACTCAAAGAAGGCCACAATC
*m-Bax*	F: TTGCCCTCTTCTACTTTGCTAG
	R: CCATGATGGTTCTGATCAGCTC
*m-β-actin*	F: CTACCTCATGAAGATCCTGACC
	R: CACAGCTTCTCTTTGATGTCAC
*p-ATF4*	F: GATCCTCCTGGAGAGAAGGTGGTAG
	R: CCGAGTGGCTGCTGTCTTGTTC
*P-CHOP*	F: TCTGGCTTGGCTGACTGAGGAG
	R: TTTCCGTTTCCTGGGTCTTCTTTGG
*p-HSP70*	F: CAACAAGATCACCATCACCAAC
	R: ACCCTTAAGGAGCTTATTGAGG
*p-Bcl-2*	F: TCGCCCTGTGGATGACTGAGTAC
	R: CCTTCAGAGACAGCCAGGAGAAATC
*p-Bax*	F: GCTTCAGGGTTTCATCCAGGATCG
	R: ACTCGCTCAACTTCTTGGTAGATGC
*p-β-actin*	F: CTACCTCATGAAGATCCTGACC
	R: CACAGCTTCTCTTTGATGTCAC

## Data Availability

The data will be made available upon request.
